# Sensitivity to Dietary Wheat Gluten in Atlantic Salmon Indicated by Gene Expression Changes in Liver and Intestine

**DOI:** 10.3390/genes11111339

**Published:** 2020-11-12

**Authors:** Amritha Johny, Gerd Marit Berge, André S. Bogevik, Aleksei Krasnov, Bente Ruyter, Christiane Kruse Fæste, Tone-Kari Knutsdatter Østbye

**Affiliations:** 1 Toxinology Research Group, Norwegian Veterinary Institute, 0454 Oslo, Norway; christiane.faste@vetinst.no; 2 Nofima-Norwegian Institute of Food, Fisheries and Aquaculture Research, 6600 Sunndalsøra, Norway; gerd.berge@nofima.no; 3 Nofima-Norwegian Institute of Food, Fisheries and Aquaculture Research, 5141 Fyllingsdalen, Norway; andre.bogevik@nofima.no; 4 Nofima-Norwegian Institute of Food, Fisheries and Aquaculture Research, 1430 Ås, Norway; aleksei.krasnov@nofima.no (A.K.); bente.ruyter@nofima.no (B.R.); tone-kari.ostbye@nofima.no (T.-K.K.Ø.)

**Keywords:** *Salmo salar*, fishmeal, intestine, liver, plant-based feed ingredients, wheat gluten, gene expression

## Abstract

Feed safety is a necessity for animal health and welfare as well as prerequisite for food safety and human health. Wheat gluten (WG) is considered as a valuable protein source in fish feed due to its suitability as a feed binder, high digestibility, good amino acid profile, energy density and most importantly, due to its relatively low level of anti-nutritional factors (ANFs). The main aim of this study was to identify the impact of dietary WG on salmon health by analysing growth, feed efficiency and the hepatic and intestinal transcriptomes. The fish were fed either control diet with fishmeal (FM) as the only source of protein or diets, where 15% or 30% of the FM were replaced by WG. The fish had a mean initial weight of 223 g and approximately doubled their weight during the 9-week experiment. Salmon fed on 30% WG showed reduced feed intake compared to the 15% and FM fed groups. The liver was the less affected organ but fat content and activities of the liver health markers in plasma increased with the inclusion level of WG in the diet. Gene expression analysis showed significant changes in both, intestine and liver of fish fed with 30% WG. Especially noticeable were changes in the lipid metabolism, in particular in relation to the intestinal lipoprotein transport and sterol metabolism. Moreover, the intestinal transcriptome of WG-fed fish showed shifts in the expression of a large number of genes responsible for immunity and tissue structure and integrity. These observations implied that the fish receiving WG-containing diet were undergoing nutritional stress. Overall, the study provided evidence that a high dietary level of WG can have a negative impact on the intestinal and liver health of salmon with symptoms similar to gluten sensitivity in humans.

## 1. Introduction

The world population is currently 7.8 billion and expected to increase by 2 billion by 2050. Between 1961 and 2016, the population growth (3.2%) outpaced the food production (1.6%) and also exceeded the total meat production (2.8%) [1]. However, the fish consumption is increasing constantly at an average rate of 1.5% per year, and the UN estimate that one in five persons depends on fish as the primary source of protein. This tremendous growth depends directly on the availability of feed resources. Atlantic salmon (*Salmo salar*) is a commercially important species [1], with the major share produced in Norway, even increasing in export volume by 4% from 2019 to 2020 [2]. Fish consumption is expected to increase in the coming years to meet the demands of the growing population. Fish is an important part of the human diet, contributing with essential amino acids, omega-3 long chain polyunsaturated fatty acids such as eicosapentaenoic acid and docosahexaenoic acid, essential minerals (Ca, P, Zn, Fe, Se, I), and vitamins (A, B, D) [3]. Growth in aquaculture is necessarily accompanied by an increase in feed production, and consequently by a need for alternative feed ingredients. The inclusion rates of fishmeal (FM) and fish oil (FO) in the diets of Atlantic salmon have decreased from 90% in 1990 to 14.5% in 2016 [4,5]. The shift to plant-based feed ingredients is a direct consequence of the reduced global availability of FM and FO. Moreover, a rising market pressure to improve the sustainability of fish farming has encouraged this development [6] and this caused the marine protein dependency ratio to decrease from 3.8 kg (1990) for 1 kg of salmon to 0.7 (2013), along with a parallel decrease in the use of marine oils [4].

Novel protein sources, e.g., from insects, are commercially available, but plant-based materials continue to be the prevalent replacement for marine ingredients. Mostly used are soybean protein concentrate (SPC), wheat and wheat gluten (WG), along with corn, faba beans, sunflower meal, pea protein concentrate, and other vegetable proteins [5]. The optimal growth of Atlantic salmon depends not only on the fish genetic profiles, rearing conditions, and diet composition but also on the feed formulation and processing [7]. Feed processing has a significant impact on the nutritional quality and digestible energy of the complete diet [8]. It has been shown that soybean meal can cause enteritis in salmon, whereas alcohol-extracted SPC appears not to affect the growth and intestinal integrity, and even enhances weight gain better than FM [9]. However, some studies have indicated that also protein concentrates from soybean and pea can trigger moderate changes in the intestine of salmon, while comparable effects were not reported for WG [10]. WG is a good pellet binder in extruded diets. It is highly digestible [11] and can replace up to 35% of FM in salmonid diets without significant negative effects [12], so that it has become widely accepted as ingredient in fish diets. However, higher levels can only be incorporated after adequate supplementation with limiting amino acids, especially lysine.

Inclusion of plant ingredients pose a potential threat to fish due to the presence of various undesirable substances including anti-nutritional factors (ANFs) such as phytoestrogens and mycotoxins as well as chemical contaminants, which can interfere with nutrient digestibility, absorption and utilisation, and negatively affect growth and health [13]. Undesirable substances may be present in feed at low levels and thus remain unnoticed due to lack of analysis, methods with insufficient detection limits, or the non-availability of reference standards, yet their presence alone or in combination may have negative implications. Dietary exposure to harmful contaminants can be detected by transcriptomic analysis showing affected pathways. WG contains comparably less ANF than protein concentrates from legumes [11]. However, major protein fractions in WG, i.e., the gliadins and glutenins [14], are associated to a range of intestinal health effects and disorders in humans [15]. So far, sensitivity to wheat gluten has not been reported in fish.

Many proteins of different origins are assessed as novel ingredients in fish feed as replacement for FM, making thorough evaluation using multidisciplinary approaches for studying effects of on fish metabolism, health, and growth performance necessary. The present study was designed to identify the impact of WG on growth, feed efficiency, metabolism, and intestinal health in Atlantic salmon. WG was administered at two inclusion levels (15% or 30%) in the diet and compared to a diet containing only FM as the protein source. Blood parameters and liver fat were analysed to identify potential health effects. Gene expression profiling by microarray analysis was performed to study the response in two metabolic important tissues, intestine and liver.

## 2. Materials and Methods

### 2.1. Preparation of Customised Salmon Diets

The fish diets were prepared at Nofima Feed Technology Centre, Fyllingsdalen, Norway by incorporating WG as the plant protein source, replacing 15% or 30% of the fishmeal (FM). The feed materials were purchased from the commercial companies Norsildmel AS (FM) (Bergen, Norway) and Tereos Syral (WG) (Marckolsheim, France). The plant ingredients used were of good quality and contained only low levels of undesirable substances such as mycotoxins, often below the limit of detection [16]. Therefore, we assumed that any change in the gene expression in different salmon tissues was not caused by these contaminants, but was a direct consequence of the presence of WG in the diet. The manufacturing process of the experimental diets for salmon has been reported previously in detail [16]. Diet formulations and compositions are given in Table 1. The fish diets were formulated in such a way that contents of total proteins, dry matter, lipids, and energy were approximately the same for all diet groups. The pellet size was adjusted to the size of the on-growing salmon. All non-oil ingredients were mixed, extruded, dried, and coated with oil. Yttrium oxide was included at 0.01% as an indigestible inert marker to quantify apparent nutrient digestibility. The diets were not balanced for amino acids and the other nutrients.

### 2.2. Fish and Feeding Trial

The experimental set-up, feeding trial, and sampling have been previously explained in detail [16]. Briefly, one-year-old post-smolt Atlantic salmon with a mean weight of 223 g were used in a nine-week feeding trial at the Nofima’s Research Station, Sunndalsøra, Norway. The experimental groups consisted of a control group receiving diet with FM as the only protein source, and two groups fed with WG-containing diets with, respectively, 15% or 30% replacement of FM. Fish were randomly distributed to the tanks (1 m^3^; *n* = 30 fish per tank, except one tank of the FM group, which had 29 fish) supplied with seawater, using three replicate tanks for each of the diet groups. Bulk weight of the fish per tank was recorded at the start of the experiment. Excess feed was collected daily from the tanks for the calculation of feed intake [17]. Fish health, feed intake, and overall welfare were regularly monitored. The water temperature was maintained at an average of 10.6 (±0.6) °C. The oxygen level at the tank outlets was higher than 90% at study start and about 80% at study end. The water flow in each tank was set to 20 L/min.

At the end of the trial, the fish were anaesthetised with a standard dose of tricaine methanesulfonate MS222 (Sigma-Aldrich, St. Louis, MO, USA), transferred to a smaller tank and euthanised with a lethal dose of 200 mg/L of the same chemical. Individual weights, liver weights, and lengths were recorded for the sampled fish (*n* = 5 fish per tank, i.e., in total 15 for each of the diets) and the rest weighed in bulk. Tissues and blood samples were taken. The sampled fish were opened and the mid-intestine was dissected out and digesta removed. The intestines were rinsed clean with phosphate-buffered saline (PBS) pH 7.4 and snap-frozen in liquid nitrogen. Livers were removed, weighed, and cut into small pieces of 1 cm before snap-freezing in liquid nitrogen. The liver and intestine samples were stored at −80 °C until analysis.

Faecal samples were collected, pooled per tank, and stored at −20 °C prior to analysis for calculating apparent digestibility coefficients (ADC). The ADC of lipid, nitrogen, and energy in the experimental diets were determined by using the equation $\mathrm{ADC}\;\left(\%\right)=\;100\;–\;100\;\times \;\left(Y_{d}\;\times {\;N}_{f}\right)\;/\;\left(Y_{f}\;\times {\;N}_{d}\right)$, where d is for diet, f for faeces, Y for yttrium content, and N for nutrient content. The growth rates were calculated according to the equation for the specific growth rate $\mathrm{SGR}\;\left(\%\right)=\left(\ln W_{1}-\ln W_{0}\right)/d)\times 100$, where W_1_ is the final weight, W_0_ the initial weight, and d are the days in experiment, and for the thermal growth coefficient TGC=1000×(W113−W013)/ddg, where ddg represents degree-days, i.e., the product of the water temperature (°C) and the number of days at this temperature in the experiment. Furthermore, the feed conversion ratio (FCR = feed consumed/biomass increase), condition factor (CF = (fish weight (g)/fish fork length (cm) × 100), and hepatosomatic index (HSI = 100 × (liver weight/total fish weight) were calculated.

### 2.3. Measurements of Blood Parameters

Blood samples were centrifuged for serum preparations, and free fatty acids (FFA), total protein (Tprot), triglycerides (TG), alanine aminotransferase (ALT), and aspartate aminotransferase (AST) were analysed at the Central Laboratory of the Norwegian University of Life Science (NMBU), Oslo, Norway.

### 2.4. Chemical Analysis of Liver Fat

Total lipids were extracted as previously described [18]. Briefly, 0.4 g liver samples of five fish in a tank were pooled, homogenised in chloroform/methanol (2:1, *v*/*v*), filtered, washed in isotonic saline, and quantified gravimetrically.

### 2.5. RNA Extraction

Total RNA was extracted from the mid-intestine and liver samples (≈10 mg) using a Biomek 4000 Automated Workstation (Beckman Coulter, Indianapolis, IN, USA), applying the Agencourt^®^ RNAdvance tissue kit (Agencourt Bioscience Corporation, Beverly, MA, USA) according to the manufacturer’s instructions: the tissues (10 mg) were transferred into 1.2 mL collection microtubes (QIAGEN, Venio, The Netherlands) with 3 mm magnetic beads, 400 µL lysis buffer, and 1 mg proteinase K. The samples were homogenised in a tissue lyser (180 s 1800 rpm; FastPrep-96, Beckman Coulter), centrifuged (1 min, 1600 rpm, Avanti™ J-301, Beckman Coulter), and placed in a heated cabinet (Termaks, Bergen, Norway) for 25 min or more until the tissue samples were completely lysed. Subsequently, the samples were processed in the Biomek 4000 workstation. RNA concentration and quality were determined using a NanoDrop 8000 Spectrophotometer (Thermo Scientific, Bremen, Germany) and an Agilent 2100 Bioanalyzer instrument with Agilent RNA 6000 Nano Kit (Agilent Technologies, Santa Clara, CA, USA) in accordance to the respective instrument protocols. An RNA integrity number (RIN) value above 7.5 was considered as satisfactory. The RNA samples were stored at −80 °C until use.

### 2.6. Microarray

Nofima’s genome-wide Atlantic salmon oligonucleotide microarray with 4 × 44k 60-mer probes, manufactured by Agilent Technologies (Santa Clara, CA, USA), was used [19]. All reagents and equipment were purchased from the same source. Two fish from each replicate tank, in total six fish per dietary group for both intestine and liver, were selected for the microarray analysis. Amplification and labelling of the total RNA (200 ng/reaction) with cyanine 3-cytidine triphosphate (CTP) was performed with the Low-Input Quick-Amp Labeling Kit (Santa Clara, CA, USA). cRNA was quantified using the NanoDrop 8000 Spectrophotometer. The Gene Expression Hybridization Kit was used for fragmentation of the labelled RNA. The samples were immediately loaded onto the microarray for hybridisation for 17 h in a hybridisation oven at 65 °C with a rotation speed of 10 rpm. The arrays were then washed for 1 min at room temperature with Gene Expression Wash Buffer I and for 1 min with Gene Expression Wash Buffer II, the latter was prewarmed and kept at 37 °C until use. The microarrays were scanned with a SureScan Microarray Scanner from the same manufacturer.

Gene expression data were processed and analysed with Nofima’s bioinformatics database STARS (Salmon and Trout Annotated Reference Sequences) [19]. Global normalisation was performed by equalising the mean intensities of all microarrays. The normalised values for the individual features were then divided by the mean value of all samples, thus determining the specific expression ratios (ER). Since the expression changes in the WG-containing diets were correlated, just the scale being greater for the WG30 diet, the results were combined for further analysis. Finally, log_2_-ER were calculated and normalised by locally weighted nonlinear regression (Lowess).

### 2.7. Data Analysis

The growth rates and other parameters (body weights, feed intake, FCR, and ADC) of salmon fed with different diets were recorded for each dietary group (considering the means of three replicate tanks, *n* = 3) and statistically tested by one-way ANOVA to assess the effects of the diets. For comparison of the individual measured parameters from the final sampling (HSI and CF), a nested mixed model was used with diet as a fixed variable and tank as a random variable, and by using tank within diet as error term for testing. Statistical differences between the dietary groups with respect to the blood parameters and liver fat contents were evaluated by one-way ANOVA followed by Duncan’s multiple range test. In microarray analysis, differently expressed genes (DEGs) were selected by difference from control (>1.75-fold and *p* < 0.05). Enrichment of GO terms (Gene Ontology) and KEGG pathways (Kyoto Encyclopedia of Genes and Genomes) was assessed with Yates’ corrected chi-square test.

### 2.8. Ethical Statement

The study was performed in compliance with the laws regulating experimentation with live animals in Norway and the experimental protocol was approved by the Norwegian Animal Research Authority (Forsøksdyrutvalget). The methods were carried out in accordance with the relevant guidelines and regulations by EU (2010/63/EU) [20]. The experiment was considered as not requiring a specific license from Norwegian Food Safety Authority as the fish received uncontaminated feed, were not subjected to experimental treatments, had not been exposed to any pain or distress, and were euthanised by an ethically approved method.

## 3. Results

### 3.1. Growth Performance

Growth performance was significantly affected by the WG diets (Table 2). Increasing inclusion of WG in the diet resulted in reduced feed intake, with the WG30-fed salmon showing significantly lower feed intake than the FM group. The WG30 group had the lowest final weight but was not significantly different from the other groups. There was no difference in growth rates (specific growth rate (SGR) and thermal growth coefficient (TGC)) between the WG15 and FM groups, whereas the WG30 group had lower SGR and TGC than the other groups. There was no measurable effect of the different diets on the condition factor (CF), ranging from 1.30 to 1.35, indicating the overall good condition of the salmon in the feeding trial. Feed conversion ratios (FCR) also did not differ between groups (Table 3). In contrast, there was a clear effect of the diets on the apparent digestibility coefficients (ADC) for nitrogen, which were higher in both WG groups than in the FM group. The ADC for energy and lipids were not different between the groups (Table 3). No mortality was observed during the feeding trial.

### 3.2. Blood Serum Analysis

The diets had a significant effect on several of the measured serum parameters (free fatty acids (FFA), total protein (Tprot), triglyceride (TG), alanine aminotransferase (ALT) and aspartate aminotransferase (AST)) (Table 4). The FFA levels were lower in both WG groups as compared to the FM-fed control group. However, the WG30 group showed higher levels of Tprot, and the liver enzyme (ALT) than both the WG15 and FM group. Moreover, there was a strong trend towards higher AST levels in the WG30 group.

### 3.3. Hepatosomatic Index and Liver Fat Content

The fat contents in the salmon livers were within the normal range of 4.9–6.9% (Table 2). There was, however, a significant dietary effect with increasing WG in the diets leading to higher liver fat percentages. The hepatosomatic index (HSI) in the WG30 group was significantly higher than in the other groups, ranging from 1.21 in the FM to 1.64 in the WG30 group.

### 3.4. Gene Expression Profiling

**Figure 1:**
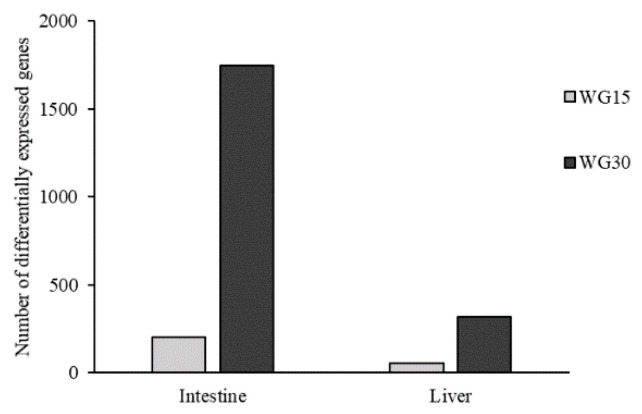
Differentially expressed genes (DEGs) in the intestine and liver of salmon fed with WG-containing diets (15% (WG15) or 30% (WG30)) in comparison to the FM control group (*n* = 6).

**Figure 2:**
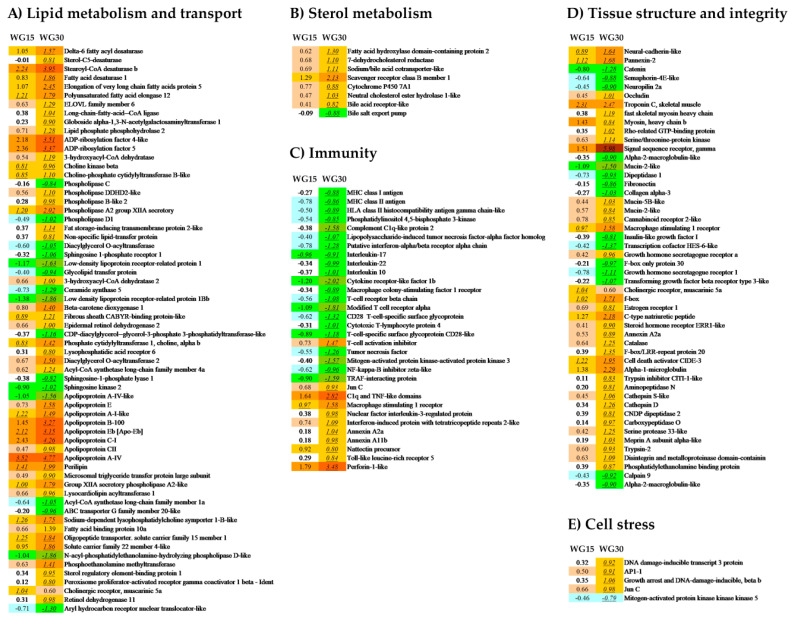
Major differentially expressed genes (DEGs) in the intestine of salmon fed with WG containing diets; genes are related to (**A**) lipid metabolism and transport, (**B**) sterol metabolism, (**C**) immunity, (**D**) tissue structure and integrity, (**E**) cell stress. Data are folds to the FM-fed control group, DEGs (>1.754-fold, *p* < 0.05) are indicated with underlined italics.

**Figure 3:**
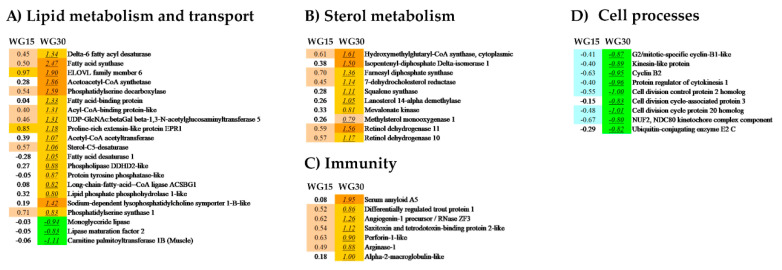
Major differentially expressed genes (DEGs) in the liver of salmon fed with WG containing diets; genes are related to (**A**) lipid metabolism, (**B**) sterol metabolism, (**C**) immunity, (**D**) cell processes. Data are folds to the FM-fed control group, DEGs (>1.754-fold, *p* < 0.05) are indicated with underlined italics.

The numbers of genes, which fulfilled the criteria for differential expression (>1.754-fold, *p* < 0.05) in the WG-fed groups, when compared to the FM group, were 204 and 1748 in intestine and 55 and 318 in liver for, respectively, the WG15 and WG30 group (Figure 1). Thus, the group showing the highest number of DEGs and the largest fold change in gene expression was in the salmon fed with 30% of WG. Of the two tissues studied, intestine was more affected by WG inclusion in the diet than the liver. The major pathways that have been altered in the intestinal transcriptome included metabolic processes related to lipid metabolism and transport, sterol metabolism, immunity, tissue structure and integrity, and cell stress (Table 5 and Table 6; Figure 2A–E). In salmon liver, processes linked to lipid metabolism and sterol metabolism were largely affected, along with a few genes related to immunity and cell-processes (Table 5 and Table 6; Figure 3A–D). Gene expression was generally more upregulated in the WG30 than in the WG15 group, both in intensity and number of DEGs in intestine and liver.

### 3.5. Intestinal Gene Expression

The partial inclusion of WG in the diets induced significant changes in the expression of genes associated with lipid metabolism (Figure 2A). In general, genes related to (a) lipoprotein assembly (*choline-phosphate cytidyltransferase* (*pcyt1B*), *sodium-dependent lysophosphatidylcholine symporter 1-B-like genes* (*msfd2a*), *choline kinase* (*chkb*), *CDP-diacylglycerol-glycerol-3-phosphate 3-phosphatidyltransferase-like gene* (*pgs1*), *N-acyl-phosphatidylethanolamine-hydrolysing phospholipase D-like* (*nape-pld*), *phosphoethanolamine methyltransferase* (*pemt*), *apolipoproteins* (*apo*), *perilipins* (*plin*), *microsomal triglyceride transfer protein* (*mtp*)), (b) fatty acid synthesis (*fatty acid desaturase* (*fads5*), *elongation of very long chain fatty acids protein* (*elovl4*, *elov5*, *elov6*), *long-chain-fatty-acid-CoA ligase* (*acsbg1*), *sterol-C5-desaturase* (*sc5d*)), *diacylglycerol O-acyltransferase 2* (*dgat2*), *phospholipase* (*ddhd2*)), and (c) fatty acid transport (*fatty acid binding protein* (*fabp7*)), along with the associated transcription cofactors (*sterol regulatory element-binding protein 1* (*srebp1*) *and peroxisome proliferator-activated receptor γ* (*pparγ*)) were the most affected in the salmon intestine.

Inclusion of WG in the diets altered genes involved in choline-based phospholipid synthesis including *pcyt1B* and *msfd2a*, involved in lipoprotein assembly, and *chkb*, which catalyses the first reaction in choline pathway for phosphatidylcholine biosynthesis. Several genes of the same pathway were downregulated in the WG30 group, including *nape-pld* and *pgs1*. Furthermore, *pemt* was upregulated in the WG30, but not in WG15 group.

The *apo* genes (*apoA-IV*, *apoEB*, *apoB-100*, *apoC1*, *apoCII*, *apoE*), which play a key role in transporting lipids from intestine and liver to peripheral tissues, were upregulated in the WG30 and to a lesser extent in the WG15 group. The lipid droplet-associated protein, *plin2* showed increased expression with both WG diets in comparison to the FM diet. Likewise, upregulation of *mtp* involved in the transport of glyceride, cholesterol ester, and phospholipids, was observed. Several other genes involved in fatty acid and TG synthesis such as *srebp1*, *fabp7*, *fads5*, *elovl4*, *elov5*, *elov6*, *acsbg1*, *sc5d*, *dgat2*, and *ddhd2* were also differentially expressed, with the highest expression found in the WG30 group. A number of genes involved in vesicle formation and transport including *ADP-ribosylation factors* (*arf*) were significantly upregulated with the WG-containing diets, with the highest increase in the WG30 group.

Sterol or cholesterol metabolism-related genes (Figure 2B) including fatty acid hydroxylase (faxdc2), sodium/bile acid cotransporter-like genes (*slc*), neutral cholesterol ester hydrolase (nceh1), dehydro cholesterol reductase (dhcr7) and bile acid receptors were upregulated in the WG30 dietary group. Furthermore, cholesterol 7-α-monooxygenase-like (cp7a1), which is involved in the conversion of cholesterol into 7-α-hydroxycholestrol, was upregulated in both WG groups. Some genes from the same group were downregulated including bile salt export pump (abcb11a). Scavenger receptor class B member (scarb1), which is a receptor for different phospholipid ligands, and cholecystokinin (cck), were also upregulated in both WG groups as compared to the FM group.

Expression of immunity-related genes (Figure 2C) indicated a hypo-immune status of fish fed a high level of WG. Various classes of pro-inflammatory and anti-inflammatory genes responsible for antigen presentation, inflammation, and overall immune response were differentially regulated in the intestine. *MHC class I and II histocompatibility antigens* (*h2-q10*), *NF-kappa-B inhibitor zeta-like* (*nfkbiz*), *interleukins* (*il17*, *il22*, *il10*), *TNF receptor-associated factor* (*traf2*), *T-cell specific surface glycoprotein* (*cd28*), *cytotoxic T-lymphocyte-protein 4-like gene* (*ctla4*) along with several others were downregulated in the WG30 group, whereas *annexins* (*anxa2*, *anxb11*), *nuclear factor interleukin-3-regulated protein* (*nfil3*), *complement C1q-like protein 2* (*ciql2*) *and TNF-like domains*, *nattectin precursor* (*natte*), and *toll like receptors* (*tlr5*) were upregulated. *Macrophage stimulating receptor* (*mst1r*) was upregulated in both WG groups.

Dietary content of WG affected the expression of several genes related to the maintenance of tissue structure and integrity (Figure 2D). Differential regulation of genes for mucosal proteins (*mucin-2*, *mucin 5b*) along with strong downregulation of genes for extracellular matrix components (*fibronectins*, *collagen α-3-(IV)* (*col4a3*), *neuropilin*, *catenin*, *semaphorin 4E*, *transcription cofactor HES-6 like* (*hes6*), and *F-box genes* (*fbxo30*), and upregulation of genes involved in various cellular processes (*occludin*, *Rho GTPase activating proteins*, *myosin* and *cyclin-dependent kinase inhibitor 1B* (*cdkn1b*)) was observed in the WG30 group. A suite of proteases and protease inhibitors were upregulated in the intestine of WG30-fed salmon (*α-1-microglobulins* (*a1m*), *trypsin inhibitor ClTI-1-like* (*citi1*), *serine proteases* (*prss33*), *meprin A* (*mep1a*) and *cathepsin S* (*ctss*)), whereas *calpain* (*capn1*) and *α-2-macroglobulin-like* genes (*a2ml*) were downregulated.

Compared to the FM group, the WG groups also showed increased expression of several stress-related genes (Figure 2E) (*growth arrest and DNA damage inducible*, *β* (*gadd45b*), *junC* and *DNA damage inducible protein* (*ddi3*)).

### 3.6. Hepatic Gene Expression

Genes involved in lipid metabolism, sterol metabolism, and immunity were significantly upregulated in the livers of WG30-fed salmon. Among the upregulated genes associated with lipid- and sterol-metabolism (Figure 3A,B) were *fatty acid synthase* (*fasn*), *acetoacetyl-CoA synthetase* (*aacs*), *phosphatidyl serine synthase* (*ptdss1*), *β-1*,*3-N-acetylglucosaminyltransferase* (*b3gnt5*), *retinol dehydrogenase* (*rdh10*, *rdh11*), *hydroxymethylglutaryl-CoA synthase*, *cytoplasmic* (*hmgcs1*), *isopentenyl-diphosphate delta-isomerase-1* (*idi1*), *farnesyl diphosphate synthase* (*fdps*), *7-dehydrocholesterol reductase* (*dhcr7*) as well as several genes functionally related to DEGs of the intestine (*mfsd2a*, *fads5*, *fads6*, *elov6*, *fabp*, *sc5d*, and *ddhd2*). The few downregulated genes in liver included *lipase maturation factor 2* (*lmf2*), *nape-pld*, *monoglyceride lipase* (*mgl*), and *carnitine palmitoyltransferase 1* (*cpt1*). The upregulated immunity-related genes (Figure 3C) in the WG30 group (*serum amyloid A5* (*saa5*), *angiogenin-1 precursor* (*ang1*), *phospholipase A2-inhibitor* (*pla2-inhibitor*), *saxitoxin and tetrodotoxin-binding protein 2-like* (*psbp*), *perforin-1-like* (*prf1*), *arginase-1* (*arg1*), *α-2-macroglobulin* (*a2m*)) are involved in inflammation. Several genes connected to cell cycle including *cell division cycle-associated protein 3* (*cdca3*), *cyclinB2 (ccnb2*), and *G2/mitotic-specific cyclin B1-like* (*ccnb1-like*) were downregulated in the WG30 group (Figure 3D).

## 4. Discussion

The present study reports the effects of the dietary inclusion of the plant ingredient WG on the growth, metabolism, and overall health of Atlantic salmon. In agreement with previous studies, there were no major effects on the digestibility and growth rate of fish fed with diet at moderate inclusion (15%) of WG [12,21,22]. There was, however, a significant reduction of the feed intake in the WG30 group, resulting in significantly lower growth rate and a tendency towards a decreasing final weight with the increased inclusion of WG. The lower feed intake is in agreement with the observed upregulation of cholecystokinin genes *cck* in both WG groups, most pronounced in the WG30 group. Cholecystokinin is a hormone involved in the regulation of food intake and satiation and regulates the digestion of fat and protein [23]. The differential expression of these genes as well as the growth hormone secretagogue receptors (*ghsr-a*: *motilin receptor* and *ghsr-1: ghrelin*) indicate an effect of gluten in the diets on appetite regulation by signalling satiation that will reduce feed intake in the salmon. The increased expression of *cck* might be caused by a gluten-induced metabolic disorder in the intestine. The lower feed intake in the WG groups might thus be explained by an imbalance in the intestine caused by low tolerance for gluten in salmon, which has not been reported previously. This is in contradiction to previous studies showing no signs of intestinal pathology in salmon fed with up to 35% crude WG protein [12] or with wheat meal [24]. A comprehensive transcriptomic analysis of effects in salmon liver and intestine connected to the dietary inclusion of WG has not been performed so far. However, changes in the expression of genes associated to muscle growth, function, metabolism, and homeostasis from exposure to WG30 have been recently determined in the fast muscle of salmon and zebrafish in a different activity of the present project [25].

Our findings with regard to a supposed gluten sensitivity in salmon were further supported by the increased expression of genes involved in lipid metabolism and transport in fish receiving the WG-containing diets, indicating a compensatory response in the intestine and problems with the transport of lipids from the intestine to the blood circulation. Similar responses were observed in the liver, however to a lower extent, comparable to observations in previous studies [26,27]. Changes in lipid metabolism and transport, steroid biosynthesis, and protein synthesis by dietary inclusion of plant proteins in fish have been reported in a number of studies [27,28,29].

In several vertebrate species, reduced tolerance to gluten can cause intestinal inflammation and malabsorption syndromes [30]. In our study, high dietary levels of WG resulted in similar symptoms of intestinal lipid malabsorption as previously described for choline deficiency in Atlantic salmon [27]. Fishmeal is the main source of choline in salmon diets. Since the customised diet in the present study had a relatively high level of FM varying from 33.4% to 63.4%, it was expected to cover the requirement for phospholipids and choline. We therefore think that the observed upregulation of several genes involved in the choline pathway (*chka*, *chkb*, *pcyt1b*, *pmt2*, *mfsd2a*, *pgs1*) is not a result of choline deficiency but probably a response to an imbalance in the synthesis of lipoproteins that are involved in intestinal lipid transport. Similar changes were found in the liver transcriptome of WG30-fed fish, showing altered expression of genes for phosphatidyl choline biosynthesis (*mfsd2a* and *ptdss1*). Thus, it can be assumed that the intestinal imbalance and metabolic disorder caused by the WG-containing diet lead also to changes in the liver metabolism.

The imbalance in the intestine induced by the WG diets is further evidenced by an upregulation in the bile acid and fatty acid metabolism, especially in the WG30 dietary group. The cholesterol biosynthetic pathway was affected in both liver and intestine of WG30-fed fish, indicating hypocholesterolaemia and an upregulated capacity for cholesterol synthesis. The observed effects are similar to those reported in previous studies on the use of plant proteins in salmon diets, where decreased lipid digestibility, reduced bile salt levels and hypocholesterolaemia were observed [31]. The detected upregulation of the squalene and lanosterol biosynthetic pathway, producing precursors of cholesterol biosynthesis, in the WG30 group could be a compensatory mechanism for low cholesterol body pools or an imbalance in the metabolism. The upregulation of markers for cholesterol biosynthesis (*dhcr7*, *srepb1*, *pparγ*, *hmgcs*) in the WG30 group indicated increased production of cholesterol. This might be a consequence of a reduced dietary level of cholesterol due to the exchange of marine proteins by plant proteins in the diet. Alternatively, impaired cholesterol and bile acid reabsorption may result from intestinal inflammation as observed in a study on soybean meal in salmon diets [28]. An imbalance in the cholesterol and bile acid absorption might also be the cause for the detected strong downregulation of intestinal ABC transporters (*abcb11*) in the WG30 group. *Abcb11* mediates the efflux of cholesterol and bile acids from liver into bile [28,32], and its mRNA expression is positively regulated by bile acid receptors (*fxr*) [33]. Although the bile acid receptor level was unaffected in the liver of the WG-fed salmon, it was upregulated in the intestine along with bile acid cotransporters.

Dietary effects on intestine and liver are connected, which we could confirm with our findings regarding WG-containing salmon. Apart from transcriptome changes, we also detected that typical markers for liver damage were elevated in the WG30 dietary group. The increase was significant for ALT and close to significant for AST. The liver fat levels were within the normal range, but were notably increased by WG dietary inclusion. The HSI of fish in the WG30 group was higher than that of the other groups. This may be caused by increased fat retention or liver fatty acid synthesis, which was observed in salmon fed with mainly plant protein- and plant oil-containing diet [34]. The assumed connection between dietary WG and an increased fatty acid synthesis in our study is supported by the detected upregulation of the transcription factor *srebp1* and the lipid metabolism genes *fadsd5*, *fadsd6*, *fasn*, *elovl6*, *fabp*, *sc5d*, *b3gnt5*, *ddhd2*, and *aacs. Acetoacetyl-coA synthetase* (*aacs*) catalyses the first reaction in fatty acid metabolism and plays a major role in the lipid synthesis of triacylglycerols, phospholipids, and cholesterol esters [35]. These responses to the WG diets, in combination with the downregulation of lipid-hydrolysing lipases (*lmf2*, *nape-pld*, *mgl*) and *cpt1*, which is involved in the β-oxidation of fatty acids, might be a compensatory response to the malabsorption of lipids in the intestine [36].

The present study also showed a high upregulation of lipid transport proteins, apolipoproteins, and perilipins, supporting an imbalance in the intestine caused by gluten sensitivity-like reactions. Deficiency in the bile transport, as discussed previously, can cause lipid droplet accumulation and triggers the increase of transport-related genes [28]. Apolipoproteins are proteins, which bind to lipids and form lipoproteins and thereby act as transport vehicles [37]. These proteins play an important role in the transport of cholesterol, triglycerides, phospholipids, and fat-soluble vitamins between the intestine, liver, and peripheral tissues. We observed the upregulation of several apolipoproteins in the intestine of both WG-fed groups as compared to the FM group, indicating an accumulation of lipids in the intestine and reduced transport of lipids to the circulation. A similar effect has been reported in a previous study on salmon fed with diets containing considerable levels of plant protein and oils [38]. The increased expression of *plin2*, involved in the coating of lipid droplets, in the intestine of both WG dietary groups further supports this possible link. Our findings are, however, in contrast with a salmon study on choline deficiency, where reduced intracellular lipid levels were reflected by the suppression of *plin2* expression [27]. However, WG-containing diets seem to primarily affect the absorption and transport of lipids in the intestine.

The detected changes in several genes connected to pathways important for maintaining the intestinal balance that were induced by the WG-containing diets, especially at the high inclusion level, gave additional evidence for the proposed connection. The regulation of the genes such as *pept1*, *occludin*, *Rho GTPase activating proteins*, *myosin*, *cck*, *ghsr*, *cdkn1b*, *fibronectin*, *collagen*, *mucin*, *hes*, and *calpain* indicated a direct effect of WG on the salmon intestine. Upregulation of *pept1*, involved in peptide transport across the enterocyte membrane [39], might be a compensatory response to alterations in the intestinal membrane and increased peptide absorption. Expression of *pept1* has been shown to be affected by dietary plant protein sources in sea bream [40]. *Occludin* expression was upregulated in the WG30 group and may indicate WG-induced reorganisation or strengthening of cell junctions. A similar response was observed in a study on salmon fed with pea proteins combined with soybean saponins [41]. The *cdkn* inhibitors, linked to cell cycle progression, were induced by inclusion of WG in the diet in our study, and might be a compensatory mechanism to reduce the rapid proliferation of cells in the intestine, whereas the downregulation of *fibronectin*, *collagen*, and *mucin* genes in WG30-fed salmon might further indicate an altered intestinal integrity. The downregulation of genes belonging to extracellular matrix (*mucins* and *collagen*) and proteases (*hes* and *calpain*) involved in intestinal development and homeostasis in the WG30 group point also at a negative effect on intestinal integrity, as observed for soybean enteritis in salmon [42].

We were also able to show that WG altered the expression of many immune genes, which has been reported in studies on chronically inflamed intestine in salmon fed with plant diets [26,41,43]. We observed downregulation of the *CD28 antigen*, *ctla4*, *IL-17*, *IL-22*, T*-cell receptor-signalling pathway* and *TNF-like domains*, *NF-kB* and *macrophage stimulating receptor* genes, along with an increase in anti-inflammatory markers in the WG30 group. These findings support the assumption that salmon have a low tolerance to wheat gluten and that exposure can lead to an imbalance in the intestine with increasing inclusion of WG in the diet. Interestingly, genes related to cell apoptosis (cell death activator genes) were strongly upregulated in both WG dietary groups, and the most in the WG30 group. The upregulated *annexin A1 and annexin A2 (anxa2*, *anxb11)*, *cannabinoid 2 receptors* and *α-1-macroglobulins* (*a1m*) are inflammation inhibitors [20,36], and previous studies on humans with gluten sensitivity also showed increased plasma concentrations of these biomarkers during increased gluten intake [44]. Moreover, the upregulation of *serum amyloid A protein (SAA5)*, *phospholipase A2 inhibitor-like genes (differentially-regulated trout protein 1)*, *arginase 1*, *angionenin 1 precursor*, *pstbps*, and *a2m* supported the initiation of a gluten-caused inflammatory effect in the salmon. DEGs that are considered as proinflammatory and anti-inflammatory markers in humans with gluten sensitivity were also upregulated in the WG30-exposed salmon. Together with the observed changes in the expression of α-macroglobulins and transglutaminase 2 (TG2), this indicated the onset of intolerance towards gluten. These genes are associated with gluten sensitivity, as they play a role in host defence mechanisms, inflammation, and protease inhibition. TG2 can catalyse the deamidation of gliadin peptides and thereby creates epitopes that are recognised by gliadin-specific T-cells in the gut [45]. Their activation triggers autoimmune enteropathological responses leading to intestinal lesions and celiac disease [46]. The detected downregulation of TG2 and upregulation of α-macroglobulins in the intestine of WG30-fed salmon could indicate a physiological effort to reduce the gluten-induced stress. The potential of the WG-based diet to trigger sensitivity responses in salmon is also supported by a previous experiment conducted in zebrafish using the same feed ingredients as in this study. Increased infiltration of eosinophilic granulocytes to the intestinal lumen was revealed by histology of the mid-intestine of gluten-fed zebrafish [47]. In humans, gluten sensitivity or celiac disease has been shown to be connected to neurological dysfunctions in addition to intestinal and extra-intestinal symptoms [48]. Similarly, the WG30-fed salmon showed a differential expression of several neural genes expressed in the mid-intestine, which can affect enteric nervous system-controlled gut functions like satiety or hunger [47,49]. Further studies are needed to understand the consequences of WG-based fish diets on neural functions and gut-to-brain signal transmission in fish and the impact on fish welfare.

## 5. Conclusions

The partial inclusion of WG in salmon diets affected feed intake, growth, and metabolism in contrast to previous studies that supported the use of WG as a suitable ingredient replacing FM. The transcriptomic changes in the intestinal and liver metabolism indicated an imbalance in the intestinal integrity and function, with the intestine being more impaired than the liver. The observed effects were dose-dependent with respect to the dietary WG inclusion, showing significance for changes caused by WG30 in comparison to WG15 and the FM controls. Genes related to lipid and sterol metabolism, immunity, and intestinal integrity were the most affected, suggesting that the fish tried to maintain homeostasis and to compensate for the negative effects of the high dietary WG level. In particular, changes in the expression of genes that in humans are connected to the development of gluten sensitivity indicated a certain incompatibility of WG-containing diets in salmon.

## Figures and Tables

**Table 1:** Formulation and chemical contents of the custom-made salmon diets used in this study. The control diet contained only fishmeal (FM) as protein source, which was partly replaced by wheat gluten (WG) at 15% (WG15) and 30% (WG30) inclusion levels.

	FM (Control)	WG15	WG30
**Diet composition (g/100 g)**			
Fishmeal	63.35	48.35	33.35
Wheat	12.0	12.0	12.0
Wheat gluten *	-	15.0	30.0
Fish oil	20.0	20.0	20.0
Additives ^#^	4.65	4.65	4.65
Total protein	45.2	46.7	48.1
Total lipids	26.5	25.4	24.3
**Chemical content (% in diet)**			
Dry matter	93.6	93.9	94.1
Protein	45.2	46.7	48.1
Lipid	26.5	25.4	24.3
Ash	13.5	11.0	8.6
Energy (MJ/kg)	21.3	21.2	21.1
Yttrium	0.007	0.007	0.007

Fishmeal (FM) was purchased from Norsildmel AS (Bergen, Norway) and contained raw materials from Blue Whiting, Herring, Whitefish, and Mackerel. * Wheat gluten (WG) with the product name AMYTEX 100 or vital wheat gluten was purchased from Tereos Syral (Marckolsheim, France). ^#^ Additives: Vitamin mix (2%), Mineral mix (0.59%), Monosodiumphosphate–24% P (2%), Yttrium oxide (0.01%), Carophyll Pink–10% (0.05%).

**Table 2:** Body weights (bw) at study start and end, feed intake, feed conversion ratios (FCR), specific growth rates (SGR), and thermal growth coefficients (TGC) of Atlantic salmon (mean ± S.E.M.; *n* = 3 replicate tanks) fed with control diet (FM) or WG-containing diets (WG15, WG30). Liver fat contents, condition factors (CF), and hepatosomatic index (HSI) of sampled fish, mean ± S.E.M.; *n* = 15, five fish per three replicate tanks.

	FM	WG15	WG30	*p*-Value
Initial wt (g)	225 ± 2	219 ± 4	223 ± 0	0.31
Final wt (g)	548 ± 34	563 ± 4	513 ± 8	0.28
Feed intake (g)	8251 ± 214 ^a^	7602 ± 12 ^b^	6620 ± 108 ^c^	0.0005
FCR	0.80 ± 0.02	0.74 ± 0.01	0.76 ± 0.01	0.08
SGR (% d^−1^)	1.53 ± 0.03 ^a^	1.54 ± 0.04 ^a^	1.36 ± 0.03 ^b^	0.02
TGC	3.40 ± 0.06 ^a^	3.41 ± 0.10 ^a^	2.97 ± 0.07 ^b^	0.01
Liver fat (%)	4.9 ± 0.0 ^b^	5.6 ± 0.2 ^ab^	6.9 ± 0.7 ^a^	0.04
CF	1.31 ± 0.02	1.32 ± 0.02	1.30 ± 0.02	0.83
HSI	1.23 ± 0.03 ^b^	1.21 ± 0.02 ^b^	1.64 ± 0.09 ^a^	0.01

^a, b, c^ Significant differences (*p* < 0.05) between diets are indicated with different letters.

**Table 3:** Apparent digestibility coefficients (ADC) for lipid, nitrogen, and energy in salmon fed with FM or WG-containing diets. Data are mean ± S.E.M.; *n* = 3 replicate tanks. Significant differences (*p* < 0.05) between the dietary groups are indicated with different letters.

Digestibility	FM	WG15	WG30	*p*-Value
Lipid	97.2 ± 0.7	97.1 ± 0.2	96.8 ± 0.4	0.86
Nitrogen	86.4 ± 0.7 ^c^	89.1 ± 0.1 ^b^	91.8 ± 0.3 ^a^	0.0005
Energy	88.6 ± 0.7	88.5 ± 0.1	88.5 ± 0.5	0.97

^a, b, c^ Significant differences (*p* < 0.05) between diets are indicated with different letters.

**Table 4:** Free fatty acids (FFA), total protein (Tprot), triglycerides (TG), alanine aminotransferase (ALT), and aspartate aminotransferase (AST) levels in the serum of salmon fed with FM or WG-containing diets. Data are mean ± S.E.M. (*n* = 15; five fish per three replicate tanks). Significant differences (*p* < 0.05) between the dietary groups are indicated with different letters.

	FM	WG15	WG30	*p*-Value
FFA (mmol/L)	0.41 ± 0.02 ^a^	0.29 ± 0.01 ^b^	0.31 ± 0.02 ^b^	0.01
Tprot (g/L)	42.9 ± 1.1 ^b^	40.7 ± 1.0 ^b^	50.3 ± 1.8 ^a^	0.02
TG (mmol/L)	3.75 ± 0.22	3.48 ± 0.33	5.15 ± 0.67	0.31
ALT (U/L)	36.7 ± 2.7 ^b^	26.1 ± 2.3 ^b^	81.3 ± 17.8 ^a^	0.04
AST (U/L)	1082 ± 143	763 ± 100	3116 ± 827	0.09

^a, b^ Significant differences (*p* < 0.05) between diets are indicated with different letters.

**Table 5:** Functional GO categories of genes that were differentially expressed in mid-intestine and liver of salmon fed with the WG-containing diets in comparison to the FM controls (*n* = 6; two fish per three replicate tanks). Features represent the number of DEGs that were enriched of the genes included in the microarray platform. The significance (*p*-value) of the enrichment was assessed by the Yates’ corrected chi-square values.

GO Categories	Features	*p*-Value	GO Categories	Features	*p*-Value
***Mid-intestine***					
Antioxidant activity	13/57	<0.001	Lipid transport	25/207	<0.001
Autophagy	23/324	0.020	Lipoxygenase pathway	5/38	0.032
Cadherin binding	76/1263	0.002	Liver development	31/481	0.027
Cholesterol biosynthetic process	16/109	<0.001	Long-chain fatty-acyl-CoA biosynthetic process	11/63	<0.001
Cholesterol efflux	18/52	<0.001	MHC class II protein complex	5/24	0.001
Cholesterol homeostasis	34/250	<0.001	MHC class II protein complex binding	8/54	0.001
Cholesterol metabolic process	26/230	<0.001	Muscle organ development	25/287	0.048
Defence response	19/223	0.004	Myosin filament	15/174	0.011
Defence response to virus	39/535	0.001	Phospholipase A2 activity	11/55	<0.001
Fatty acid biosynthetic process	12/131	0.015	Phospholipid metabolic process	17/158	<0.001
Glycolipid biosynthetic process	8/51	<0.001	Retinoid metabolic process	22/196	<0.001
Immune response	59/971	0.006	Steroid binding	10/95	0.009
Innate immune response	74/1196	0.001	Sterol metabolic process	6/54	0.043
Interferon-γ-mediated signalling pathway	35/241	<0.001	Transforming growth factor β receptor binding	10/116	0.046
Iron ion binding	29/403	0.006	Triglyceride catabolic process	19/63	<0.001
Keratinisation	16/199	0.018	Triglyceride metabolic process	10/77	0.001
Linoleic acid metabolic process	12/82	<0.001	Ubiquitin protein ligase activity	45/657	0.001
Lipid catabolic process	32/235	<0.001	Ubiquitin-dependent protein catabolic process	51/865	0.022
Lipid homeostasis	18/159	<0.001	Very-low-density lipoprotein particle	10/52	<0.001
Lipid metabolic process	36/572	0.023	Wound healing	25/361	0.021
Lipid particle	26/300	<0.001	Xenobiotic metabolic process	21/228	<0.001
***Liver***					
Cholesterol biosynthetic process	12/109	12/109	Fatty acid biosynthetic process	6/131	<0.001
Iron ion binding	8/403	0.010	Lipid metabolic process	10/572	0.011
Liver development	8/481	0.040	Peptidase inhibitor activity	7/153	<0.001
Receptor-mediated endocytosis	7/394	0.039	Sterol biosynthetic process	5/35	<0.001
Triglyceride metabolic process	5/77	<0.001	Xenobiotic metabolic process	6/228	0.004

**Table 6:** KEGG pathways of genes that were differentially expressed in mid-intestine and liver of salmon fed with the WG-containing diets in comparison to the FM controls (*n* = 6; two fish per three replicate tanks). Features represent the number of DEGs that were enriched of the genes included in the microarray platform. The significance (*p*-value) of the enrichment was assessed by the Yates’ corrected chi-square values.

KEGG Pathways	Features	*p*-Value	KEGG Pathways	Features	*p*-Value
***Mid-intestine***					
α-Linolenic acid metabolism	13/32	<0.001	GnRH signalling pathway	21/288	0.020
Arachidonic acid metabolism	10/90	0.005	Linoleic acid metabolism	10/38	<0.001
Biosynthesis of unsaturated fatty acids	8/40	<0.001	mTOR signalling pathway	12/148	0.043
Ether lipid metabolism	12/64	<0.001	PPAR signalling pathway	19/164	<0.001
Fc epsilon RI signalling pathway	15/180	0.015	Retinol metabolism	18/98	<0.001
Focal adhesion	46/716	0.006	Steroid hormone biosynthesis	7/70	0.049
Glycerophospholipid metabolism	20/196	<0.001	Vascular smooth muscle contraction	29/418	0.011
***Liver***					
Arginine and proline metabolism	6/196	0.001	p53 signalling pathway	6/150	<0.001
Cell cycle	6/273	0.016	PPAR signalling pathway	6/164	<0.001
Drug metabolism—other enzymes	5/78	<0.001			
